# A prospective, randomised double-blind study on the anaesthetic effect of dexmedetomidine hydrochloride in brainstem tumour surgery

**DOI:** 10.1186/s12957-019-1654-0

**Published:** 2019-07-09

**Authors:** Sheng-Xiang Wu, Hua-Qin Chen

**Affiliations:** 1 Department of Neurology, The First People’s Hospital of LanZhou City, No.1 of Wujiayuan Street, Qilihe District, LanZhou, 730050 China; 2 Department of Endocrinology, The First People’s Hospital of LanZhou City, No.1 of Wujiayuan Street, Qilihe District, LanZhou, 730050 China

**Keywords:** Clinical application, Dexmedetomidine hydrochloride, Brainstem tumour, Effect analysis, Anaesthesia

## Abstract

**Context:**

Brainstem tumour surgery is difficult, and accidents can easily occur.

**Objective:**

To explore the effect of dexmedetomidine hydrochloride on brainstem tumour surgery.

**Design, setting and participants:**

A total of 60 patients with brainstem tumours successfully operated on by our hospital from March 2016 to March 2018 were selected as subjects.

**Interventions:**

These patients were randomised into two groups: the research group (*n* = 30) and control group (*n* = 30). Patients in the control group were given propofol together with a placebo (0.9% sodium chloride solution) to maintain anaesthesia after general anaesthesia, while patients in the research group were supplemented with dexmedetomidine hydrochloride.

**Main outcome measure:**

Awakening time, overall stability of various indicators in the operation and adverse reactions during the awakening period were observed.

**Results:**

The results revealed that patients in the research group had a longer awakening time, higher mean stability rate, higher effective rate and less incidence of adverse reactions during the awakening period than the control group; the differences were all statistically significant (*P* < 0.05).

**Conclusion:**

Dexmedetomidine hydrochloride has a good analgesic effect in intraoperative anaesthesia during brainstem tumour surgery, which significantly reduces the incidence of adverse reactions. Therefore, it can be used to assist anaesthesia during brainstem tumour operations and is worthy of clinical popularisation and application.

## Introduction

Dexmedetomidine hydrochloride can be used for general anaesthesia, local anaesthesia, spinal anaesthesia and nerve-blocking anaesthesia in clinical operations [1]. It does not cause respiratory depression and has good sedative and analgesic effects during surgery [2]. In addition, the time for patients to regain consciousness after surgeries is shorter when dexmedetomidine hydrochloride is used, which can protect the nerves [4]. Due to the small size of the brainstem and the large number of nerves, as well as their wide distribution, brainstem tumour surgery is one the most difficult and dangerous surgeries, with accidents easily occurring [4]. In recent years, treatment methods for brainstem tumours have been continually improving and the success rate of brainstem tumour surgery has significantly increased; nevertheless, efforts are still needed to reduce the injury rate in patients during brainstem tumour surgery [5, 6]. To allow patients to experience a safer, more comfortable and painless process during brainstem tumour surgeries, our hospital introduced dexmedetomidine hydrochloride to improve the quality of anaesthesia. This experiment was designed to study its effects. Details are reported as follows.

## Methods

### Study population

From March 2016 to March 2018, a total of 60 patients with brainstem tumours that were successfully operated on in our hospital were enrolled in the present study. Among these patients, 30 were male and 30 were female. The age of the patients ranged from 40 to 70 years, with an average age of 55.12 ± 1.25 years. All patients provided a signed informed consent form to participate in the present study. The present study was approved by the hospital’s ethics committee.

### Randomisation

These patients were randomly divided into two groups—the research group and control group (*n* = 30, each group)—using a randomisation table. Each patient was given a random number, and dexmedetomidine hydrochloride and its placebo (0.9% sodium chloride solution) were blind labelled so that both the patients and the investigators were blinded for the grouping of the patients. Among the patients in each group, 15 patients were male and 15 patients were female. The routine examinations before operation revealed no difference between the two groups.

### Study interventions

After general anaesthesia, patients received propofol to maintain the anaesthesia. Patients in the research group were additionally given dexmedetomidine hydrochloride for auxiliary anaesthesia on the basis of the anaesthesia in the control group, while patients in the control group received placebo instead of dexmedetomidine hydrochloride. Premedication before anaesthesia included 20% mannitol and atropine. Dexmedetomidine hydrochloride was diluted with 0.9% sodium chloride solution to a concentration of 4 μg/ml under aseptic conditions and slowly intravenously injected to patients at a dose of 1 μg/kg. The injection time was more than 10 min [3]. The injection dose was determined according to each patient’s condition and recorded. During surgery, PaCO_2_ was maintained using mechanical ventilation at 4~5 kPa (30~37 mmHg). When blood pressure and heart rate changed during surgery, the procedure was stopped until the blood pressure or heart rate returned to normal. At the awakening time, the overall stability of the indexes during the operation and adverse reactions during the awakening period were observed in the two groups.

### Study outcomes

Adverse reactions, such as restlessness, nausea, vomiting and confusion, were observed in the two groups, and the rate of adverse reactions was used to evaluate the nursing effect. The awakening time of patients in these two groups was observed, and the mean awakening time calculated. The vital signs of the patients, including heart rate, arterial pressure and blood oxygen saturation, were observed during the operation. Those patients that showed stable vital signs during the operation were considered stable patients, and thus, the mean stability rate of patients (the percentage of patients that were considered stable) in the two groups was summarised [3]. The effective rate was calculated based on the mean awakening time, mean stability rate and incidence of adverse reactions. Those patients with a long awakening time, high stability and no or mild adverse reactions were considered effective.

### Sample size and statistical analysis

Power analysis based on the effective rate showed that the minimum sample size was 20 for each group. Data were statistically analysed using the statistical software SPSS 13.0. Count data were compared using a chi-squared test (%) with Fisher’s exact test. Measurement data were compared using a *t* test (expressed as *x* ± SD). *P* < 0.05 was considered statistically significant.

## Results

From March 2016 to March 2018, a total of 60 patients with brainstem tumours that were successfully operated on in our hospital were enrolled in the present study. The incidence of adverse reactions was compared between the two groups. The nursing effect was significantly greater in the research group than in the control group (*P* < 0.05, Table 1).

**Table 1:** Comparison of the incidence of adverse reactions (one case, %)

Groups	Cases	Restlessness	Nausea and vomiting	Confusion	Incidence
Control group	30	3	2	7	40.0%
Research group	30	1	0	1	6.7%
*χ* ^2^	/	4.0294	4.191	4.325	6.935
P	/	< 0.05	< 0.05	< 0.05	< 0.05

The effective rate between these two groups was compared, and the satisfaction rate of nursing was significantly higher in the research group than in the control group (*P* < 0.05, Table 2).

**Table 2:** Comparison of the effective rate (one case, %)

Groups	Cases	Awakening time	Mean stability rate	Incidence of adverse reactions	Effective rate
Control group	30	25.50 ± 3.10	72%	40.0%	69.4%
Research group	30	36.24 ± 5.62	92%	6.7%	94.1%
*χ* ^2^	/	5.394	6.191	7.125	7.635
P	/	< 0.05	< 0.05	< 0.05	< 0.05

## Discussion

Generally, brainstem tumours are located in the midbrain, pons and medulla oblongata. Due to the complicated structure and function of the brainstem, brainstem tumour surgery in the past was a forbidden area of surgery [4]. In addition to causing damage to cranial nerves, brainstem tumours can also lead to intracranial hypertension and brain oedema, which threaten the life of patients [4]. Although brainstem tumour surgery is still very risky, the rate of intraoperative accidents can be reduced if surgical resection is only conducted for limited, nodular, or cystically degenerated and well-differentiated tumours [4]. For benign brainstem tumours, total resection is usually performed to achieve a radical resection effect, while malignant tumours are usually treated with a combination of chemotherapy and radiotherapy [6]. In traditional brainstem tumour surgery, anaesthetics usually include propofol, ropivacaine and midazolam. Although they can help to reduce the pain of patients and maintain sedation during surgery, postoperative mental confusion and consciousness delay often occur [7]. In recent years, due to medical improvements and the continuous development of microsurgical techniques, the survival rate of patients undergoing brainstem tumour resections has been significantly improved through combining it with radiotherapy, and some patients can be completely cured [6].

Dexmedetomidine hydrochloride is a newly developed anaesthetic adjuvant, which is a dextral isomer of a α2-adrenoreceptor agonist [7]. Its high selectivity to the central α2-adrenoreceptor allows it to inhibit the release of noradrenaline by acting on the excitant presynaptic membrane α2 receptor [8]. Furthermore, it can terminate the conduction of pain signals by acting on the α2 receptor in the spinal cord [9–11], thus leading to the effects of sedation and analgesia, which allows a stable calming and awakening effect during surgery [12, 13]. Therefore, patients can undergo surgery in a natural sleep state [14]. Dexmedetomidine can inhibit the sympathetic nerves by activating postsynaptic membrane receptors, which subsequently reduces blood pressure and heart rate, improves haemodynamic stability and reduces the incidence of myocardial ischemia during the operation [15, 16]. Furthermore, it also relieves the restlessness and tension of patients during the operation [17, 18]. In addition, as dexmedetomidine hydrochloride has no respiratory depression effects, when it is used in surgeries, the dosage of anaesthetics can be reduced, which protects patients’ nerves and effectively shortens the time they take to recover consciousness. This not only reduces the incidence of respiratory obstruction and laryngospasm in patients but also allows patients to cooperate with the doctor for further treatment [19–21].

The introduction of dexmedetomidine hydrochloride in the anaesthesia department of our hospital has not only improved patient comfort and increased the effective rate and safety during anaesthesia, but has also prolonged the postoperative awakening time of patients and reduced the incidence of restlessness, nausea and confusion. Hence, this has been uniformly well-received by patients and medical staff [22, 23]. In the present study, after general anaesthesia, patients in the control group received propofol together with a placebo to maintain the anaesthesia, while patients in the research group were additionally given dexmedetomidine hydrochloride for auxiliary anaesthesia on the basis of the anaesthesia in the control group. The results of the present study revealed that the incidence of postoperative adverse reactions was significantly lower in the research group than in the control group (*P* < 0.05), while the total effective rate was significantly higher in the research group than in the control group (*P* < 0.05). The high confusion rate (7/30) in the control group may be because the confusion rate after brain surgery is normally higher than after other surgeries. Due to the complex operation and amount of anaesthetic required, in brainstem tumour surgeries, the confusion rate may be even higher.

Other studies have also explored the anaesthetic effect of dexmedetomidine hydrochloride in brain surgery. For example, in Moore et al., a subject was in a lucid interval after stopping propofol and experienced agitation, which led to propofol being used again, resulting in oversedation; thus, an electroencephalogram could not be performed. After using dexmedetomidine, however, the agitation disappeared and the subject could maintain lucidity, meaning the electroencephalogram and operation could be performed [24]. Rozet et al. used dexmedetomidine when implanting deep brain stimulators in patients with Parkinson’s disease, and it was shown that dexmedetomidine effectively reduced the use of antihypertension drugs and improved patient satisfaction while still maintaining the sedation level [25].

The biggest limitation of this study is the relatively small sample size. Therefore, a study with a larger number of patients will be conducted in the future to further support the findings. In addition, the best time period to use dexmedetomidine during brainstem tumour surgery still needs to be identified.

## Conclusion

In summary, dexmedetomidine hydrochloride has good anaesthetic and analgesic effects in brainstem tumour surgery and significantly reduces the incidence of adverse reactions during the awakening period. Therefore, it can be used to assist anaesthesia during brainstem tumour operations and is worthy of clinical popularisation and application.

## Data Availability

Data are to be made available on request.
